# Sea ice, rain-on-snow and tundra reindeer nomadism in Arctic Russia

**DOI:** 10.1098/rsbl.2016.0466

**Published:** 2016-11

**Authors:** Bruce C. Forbes, Timo Kumpula, Nina Meschtyb, Roza Laptander, Marc Macias-Fauria, Pentti Zetterberg, Mariana Verdonen, Anna Skarin, Kwang-Yul Kim, Linette N. Boisvert, Julienne C. Stroeve, Annett Bartsch

**Affiliations:** 1 Arctic Centre, University of Lapland, Rovaniemi, Finland; 2 Geographical and Historical Studies, University of Eastern Finland, Joensuu, Finland; 3 Dendrochronology Laboratory, University of Eastern Finland, Joensuu, Finland; 4 School of Geography and the Environment, Oxford University, Oxford, UK; 5 Swedish University of Agricultural Sciences, Uppsala, Sweden; 6 Earth and Environmental Sciences, Seoul National University, Seoul, Republic of Korea; 7 Earth System Science Interdisciplinary Center, University of Maryland, College Park, MD, USA; 8 National Snow and Ice Data Center, University of Colorado, Boulder, CO, USA; 9 Department of Earth Sciences, University College London, London, UK; 10 Zentralanstalt für Meteorologie und Geodynamik, Vienna, Austria; 11 Geodäsie und Geoinformation, Technische Universität, Vienna, Austria

**Keywords:** *Rangifer tarandus*, Yamal Peninsula, Nenets herders, Barents and Kara seas, West Siberia, climate change

## Abstract

Sea ice loss is accelerating in the Barents and Kara Seas (BKS). Assessing potential linkages between sea ice retreat/thinning and the region's ancient and unique social–ecological systems is a pressing task. Tundra nomadism remains a vitally important livelihood for indigenous Nenets and their large reindeer herds. Warming summer air temperatures have been linked to more frequent and sustained summer high-pressure systems over West Siberia, Russia, but not to sea ice retreat. At the same time, autumn/winter rain-on-snow (ROS) events have become more frequent and intense. Here, we review evidence for autumn atmospheric warming and precipitation increases over Arctic coastal lands in proximity to BKS ice loss. Two major ROS events during November 2006 and 2013 led to massive winter reindeer mortality episodes on the Yamal Peninsula. Fieldwork with migratory herders has revealed that the ecological and socio-economic impacts from the catastrophic 2013 event will unfold for years to come. The suggested link between sea ice loss, more frequent and intense ROS events and high reindeer mortality has serious implications for the future of tundra Nenets nomadism.

## 1. Introduction

As Arctic warming has significantly exceeded that of lower latitudes in recent decades [1], indigenous peoples have reported symptoms of accelerating change, even while characterizing extreme weather events as ‘normal’ in the context of life lived on land and/or sea [2,3]. In the Eurasian Arctic, the rapid retreat and thinning of sea ice in the Barents and Kara Seas (BKS) are critical components of feedbacks to Arctic and global climate change [1,4]. Coincidentally, autumn and winter rain-on-snow (ROS) events, resulting in ice-encrusted pastures and mass starvation of semi-domesticated reindeer (*Rangifer tarandus*), have increased in frequency and intensity across the northwest Russian Arctic [5,6]. This region is home to the world's largest and most productive reindeer herds. Warmer/wetter winters have negatively affected the much smaller wild reindeer populations on High Arctic Svalbard [7,8]. In the Low Arctic, there is an urgent need to understand whether and how regional sea ice loss is driving major ROS events over mainland Russia and, crucially, the implications of such events for the region's ancient and unique social–ecological systems (SES).

At the circumpolar level, it has been proposed that increases in Arctic terrestrial primary productivity are linked to sea ice decline and thinning [9–11]. However, evidence based on tundra shrub dendroclimatology from Nenets Autonomous Okrug and Yamal-Nenets Autonomous Okrug (YNAO) does not support such a linkage in the BKS region: in recent decades, the trend of increasing deciduous shrub growth appears closely tied to more frequent and intense summer high-pressure systems over West Siberia [12]. While attention has focused on summer temperature coupled with contraction of summer reindeer pastures due to incremental hydrocarbon development [13], autumn and winter warming have been severe, leading to large changes in regional biota [6,14,15].

In particular, a major ROS event during autumn/winter 2013–2014 led to the starvation of 61 000 reindeer out of a population of *ca* 275 000 animals on the Yamal Peninsula [14]. Historically, 2013–2014 is the region's largest recorded mortality episode, and sits within a pattern of more frequent and intense autumn/winter ROS events [5,6]. If sea ice loss is driving increasingly severe ROS events and high reindeer mortality, it will have serious implications for the future of tundra Nenets nomadism. Here, we review evidence for autumn atmospheric warming and precipitation increases over Arctic coastal lands in proximity to the BKS and their links to sea ice loss. We consider how both recent (2006 and 2013) and historic ROS events have shaped Nenets strategies for coping with extreme weather from the perspective of recent literature, including modelling efforts, and empirical measures of sea ice and associated atmospheric conditions. We also explore Nenets strategies for adaptation to ROS events in recent decades and into the future.

## 2. Material and methods

Around 6000 of the approximately 30 000 indigenous Yamal Nenets of West Siberia are reindeer nomads who migrate up to 1200 km annually between lichen woodlands (winter pastures) south of the Ob River and northern shrub–graminoid tundra (spring/summer/autumn pastures) on the Yamal Peninsula [13]. The total reindeer population in YNAO was 705 000 in January 2016, of which 394 000 were privately owned. Migratory units range in size from 100 to several thousand animals. Between March 2014 and April 2016, we surveyed 60 herders and administrators in the Yamalski and Priuralski raions, representing the Yarsalinskaia and Baidaratskaia tundras (electronic supplementary material, S1). Via intensive participant observation in all seasons with Nenets nomads, we created a detailed oral history of herding strategies and movements over several decades.

We combed archives of Atmospheric InfraRed Sounder (AIRS) data to search for both seasonal and date-specific anomalies in atmospheric conditions over the BKS in two winters with high reindeer mortality (2006–2007 and 2013–2014). Archives of Advanced Scatterometer (ASCAT; electronic supplementary material, S2) data were similarly searched to detect the autumn 2013 ROS event over land. Sea ice extent was estimated using Special Sensor Microwave Imager/Sounder (SSMIS; electronic supplementary material, S3). We checked for precipitation anomalies using European Reanalysis (ERA)-Interim data, a global atmospheric reanalysis from the European Centre for Medium-Range Weather Forecasts, which provides medium-range forecast data for this region. The findings from the combined quantitative and qualitative (i.e. participatory) approaches are coupled with a literature review that focuses on modelling of BKS ice loss.

## 3. Results

**Figure 1.**
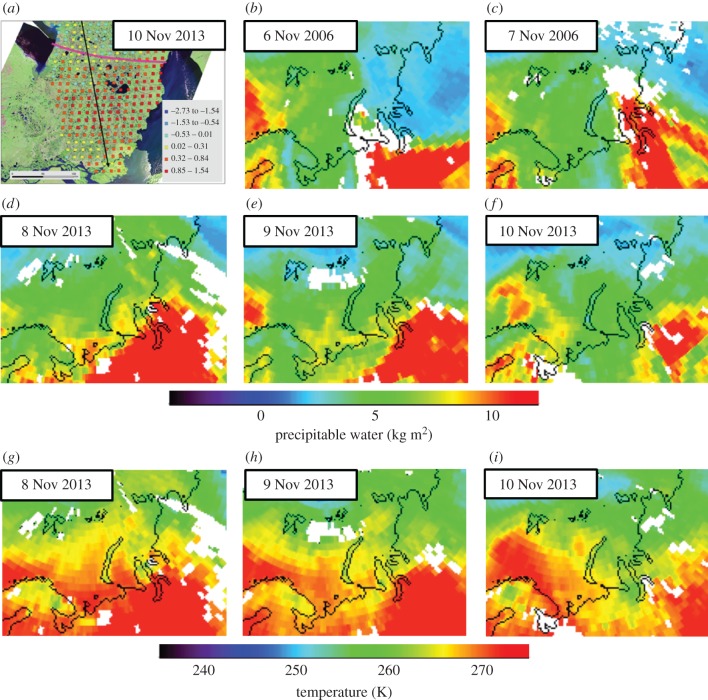
ASCAT detected backscatter difference (dB), southern Yamal Peninsula 10 November 2013. Pink line borders severely iced pasture area; black arrow indicates reindeer herders' southward migration. (*a*) AIRS daily total precipitable water from (*b–c*) 6–7 November 2006 and (*d–f*) 8–10 November 2013 and 925 hPa temperature (*g–i*) from 8–10 November 2013 for the BKS region. White indicates missing data and black outlines the coasts.

**Figure 2.**
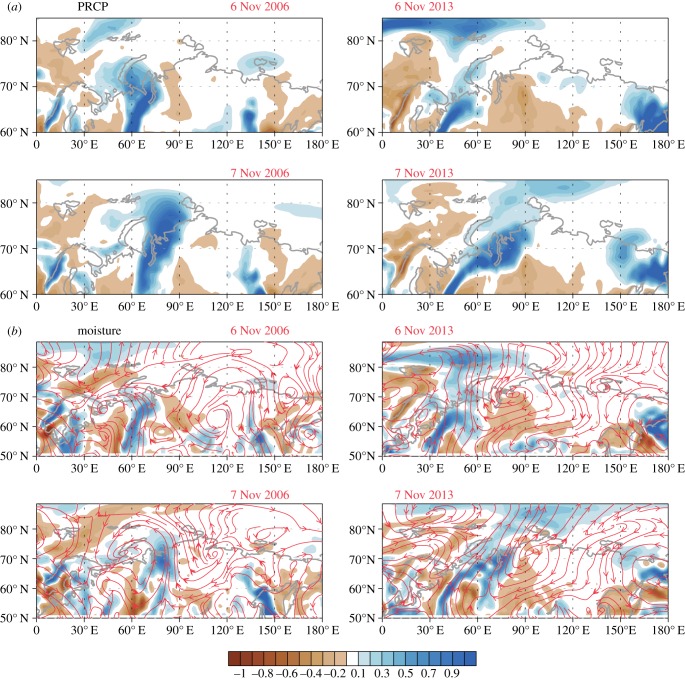
Anomalous precipitation (mm) patterns 6–7 November 2006 (left column) and 6–7 November 2013 (right column). Anomalies are to the respective monthly averages (*a*). Moisture transport (streamline) and convergence of moisture transport (shading: 10^−7^ s^−1^) in the 1000–850 hPa level in 2006 and 2013 (*b*). Moisture convergence is overall in reasonable agreement with precipitation, suggesting that convergence of increased moisture is primarily responsible for precipitation.

During the period 5–10 November 2006 (the date of the 2006–2007 winter ROS event) and 5–10 November 2013, SSMIS shows that BKS ice was steadily decreasing (electronic supplementary material, S4). Herders reported that the most recent catastrophic ROS event (*serad po* in Nenets language, i.e. *bad/distress year*) began on 8–9 November 2013 with about 24 h of rain, after which temperatures dropped and remained below freezing for the remainder of the autumn and throughout the winter. ASCAT data accurately detected the severe icing of pastures beginning 10 November 2013 over most of the southern Yamal Peninsula, an area covering *ca* 27 058 km^2^ (figure 1*a*). Autumn AIRS data indicate anomalously high total precipitable water over Nenets Okrug on 6 November 2006 and 8 November 2013, and over YNAO on 7 November 2006 and 8–9 November 2013 (figure 1*b–f*). According to AIRS data, air temperatures were anomalously high over Nenets Okrug on 6 November 2006 and YNAO on 7 November 2006. Anomalous high air temperatures characterized both regions on 6–9 November 2013, followed by cold anomalies over the Yamal Peninsula on 10 November 2013 (figure 1*g–i*). AIRS detected positive sensible heat fluxes from the surface to the atmosphere over the Barents Sea (BS) on 5–7 November 2006 and the Kara Sea (KS) on 8–9 November 2006. In 2013, positive sensible heat flux was detected over the BS on 5–8 November and the KS on 9 November. ERA-Interim data forecasted high precipitation anomalies over Nenets Okrug and YNAO on 6–7 November 2006 and 6–7 November 2013 (figure 2*a*), coupled with wind advection from the south (figure 2*b*). ERA data have close agreement with empirical data and incorporate sea ice data. By spring–summer 2014, the private herders who had lost most or all of their animals to starvation were functionally stranded in the tundra. With no draught reindeer to haul their camps, they resorted to full-time subsistence fishing and borrowed breeding stock to rebuild their herds, a multi-year process. Herders identified other historically bad icing events since World War II as occurring about once per decade, e.g. in 1947, 1954, 1974 and 1996.

## 4. Discussion

Warmer, wetter Arctic winters over the past decade have raised concern [16,17], particularly regarding impacts of ROS events on Eurasian semi-domesticated and wild reindeer populations [5,7,8,14,15,18]. Recent winter sea ice retreat is most pronounced in the BS [19]. This has been attributed to increasing ‘Atlantification’ [19,20] and has been linked to anomalous warm advection over the BS in light ice years [21]. Another factor in BKS ice loss is Ural Blocking with a positive North Atlantic oscillation (NAO), which was more persistent in 2000–2013 than in 1979–1999 [22]. Modelling efforts, such as atmospheric reanalyses, including the ERA-Interim, have indicated anomalously high winter sensible heat flux in the BS [23]. In a recent circumpolar comparison, BS was the only region with significant warming in all models [23]. Negative ice concentration anomalies were most pronounced in the BS, with significant precipitation increases over regions of winter sea ice loss, and atmospheric warming spreading to neighbouring landmasses around the BS [24]. However, a decrease of wintertime sea ice cover in the BKS does not always result in *a priori* expected warming over adjacent continental areas [25]. At the same time, quantitative links between Arctic sea ice retreat and increasing precipitation remain poorly constrained [26], yet important linkages between November BS ice loss and the NAO sign for the following winter have been reported [11].

Meteorological data show that rain fell over the central YNAO on 8–9 November 2013 [7], and on 7 November 2006, but not in southern forest–tundra and northern tundra zones. AIRS data for winter 2013–2014 (not shown) support herders' observations that cold weather prevailed after ice crust formed on 10 November 2013. ASCAT, despite low sensitivity to snow structural change relative to QuikSCAT [5], was able to capture the event owing to its intensity and severity (figure 1*a*). The ERA-Interim pattern of moisture convergence is similar to that of precipitation detected (figure 2; electronic supplementary material, S5–S9). Although wind streamline indicates that moisture is transported from the south, this does not mean that moisture originally derived from the continent, but it would have been introduced earlier from the sea (electronic supplementary material, S10).

Some of the individual elements in the chain of events that proceeds from *Warming* → *Sea ice decline* → *Increased precipitation and winter temperatures* → *ROS events* → *Reindeer mortality* → *SES resilience* have been reported elsewhere [4–9,11,13,16–24]. However, (i) this is the first time the whole, integrated picture is presented for a region where the coupling between sea ice-related environmental changes and critically important semi-domesticated reindeer nomad SES is manifest quite clearly and (ii) this is the first study to propose a link between brief but spatially significant retreat of November sea ice and massive ROS events over the Russian mainland. We stress the idea that sea ice declines are very likely linked with increased precipitation and higher temperatures in the BKS region. It is logical to infer that ROS events will be more frequent in these situations.

The pattern of more frequent and intense autumn/winter ROS events in Nenets Okrug and YNAO mirrors that of summer high-pressure systems over West Siberia. Together, the regional autumn, winter and summer warming trends present real challenges to maintaining tundra reindeer nomadism as a viable livelihood. Yet, indigenous peoples have critical data and perspectives to contribute to understanding climate change [2,3,27,28] and consciously facilitate their own social–ecological resilience through collective agency [13]. Nenets oral histories documented that smaller, more nimble privately owned herds fared better than larger collective herds. This strategy has worked well for dealing with encroaching infrastructure [13]. Regional warming already exceeds the 1.5°C scenario envisioned by the COP21 Paris agreement of 2015. Our analysis suggests that decreasing November BKS ice extent is linked to precipitation over coastal lands, putting Nenets herds and herders at risk. If BKS ice continues to decline, better forecasts of autumn ice retreat coupled with additional mobile slaughterhouses could help to buffer against reindeer starvation following future ROS events. Even a few days of early warning could make a critical difference. This could be achieved via combined use of empirical data coupled with ERA-reanalysis. Realizing mutual coexistence of tundra nomadism within the Arctic's largest natural gas complex under a warming climate will require meaningful consultation, as well as ready access to—and careful interpretation of—real-time meteorological and sea ice data and modelling.

## Supplementary Material

Supplementary Material 1

## Data accessibility

AIRS, SSMIS, ERA and MERRA-2 data are freely available, respectively, at: airs.jpl.nasa.gov, nsidc.org, apps.ecmwf.int/datasets/data/interim-full-daily and https://gmao.gsfc.nasa.gov/reanalysis/MERRA-2/.
