# Supplementary material for: Sea ice, rain-on-snow and tundra reindeer nomadism in Arctic Russia
Source: Biol Lett. 2016 Nov;12(11):20160466. doi: 10.1098/rsbl.2016.0466 (PMC5134033; doi:10.1098/rsbl.2016.0466)
Supplement: Supplementary Material 1 [file rsbl20160466supp1.docx]

**Forbes BC et al. “Rain-on-Snow and Tundra Reindeer Nomadism"**

**Supplementary Material**

**1. Interviews with Nenets reindeer herders**

The total of 60 informants encompassed adult men (ca. 70%) and women (30%), in the age cohorts >60 yrs old (40%), 50-60 yrs (40%) and <40 yrs (20%). These represented both private and collectively managed units on the territory of the Yarsalinski sovkhoz, which has the longest migration route, and the Panaevsk sovkhoz.

Discussions with herders and administrators were guided geographically employing a combination of: (1) large (1:100000) to medium (1:500000) scale topographic maps with place names; and (2) moderate (Landsat) to very high-resolution (WorldView2, GeoEye, Quickbird2) satellite imagery. Primary topics included not only the most recent ROS event and resulting massive reindeer mortality of autumn/winter 2013-14, but also the patterning of past events and nomadic responses in space and time during the last century. Secondary topics encompassed the relationship between extreme ROS events and pasture conditions, including the status of tall tundra willows (*Salix lanata*).

**2. ASCAT dataset source**

Department of Geodesy and Geoinformation, Vienna University of Technology, Gusshausstrasse 27-19, 1040 Vienna, Austria. Contact person: [annett.bartsch@tuwien.ac.at](mailto:annett.bartsch@tuwien.ac.at)

**3. SSMIS** **dataset source**

Cavalieri D, Parkinson C, Gloersen P, Zwally HJ. 1996, updated yearly. Sea Ice Concentrations from Nimbus-7 SMMR and DMSP SSM/I-SSMIS Passive Microwave Data, Version 1. [indicate subset used]. Boulder, Colorado USA: NASA DAAC at the National Snow and Ice Data Center. <http://dx.doi.org/10.5067/8GQ8LZQVL0VL>

**4. Barents and Kara Sea Ice extent (top) and concentration (bottom) from SSMIS**

The figures highlight in green the windows of significant sea ice decrease that took place in early November 2006 (black) and 2013 (red). These are shown against the long-term mean of 1981-2010 (blue).

**
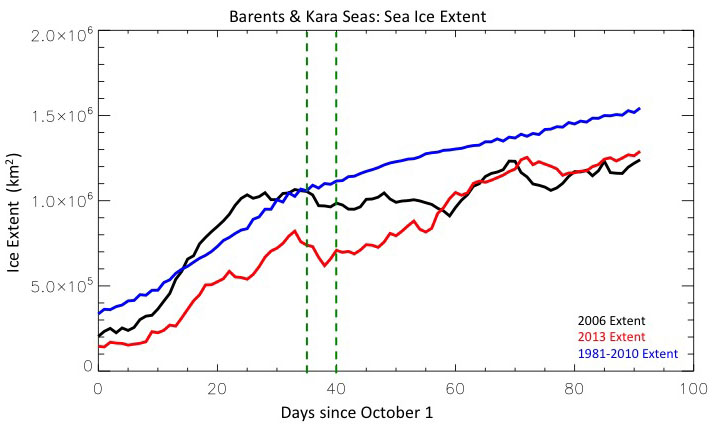
**

**
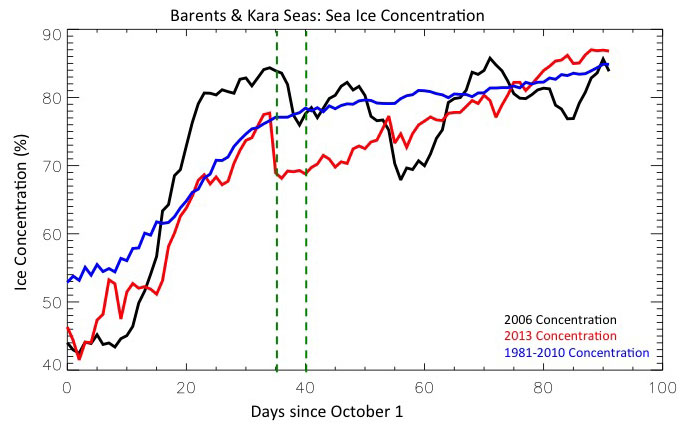
**

**5. Amplitude time series of the Arctic Amplification/Sea Ice Reduction mode.**

This index specifically measures the amount of reduction in sea ice concentration in the Barents and Kara Seas based on the ERA-interim reanalysis data. The years 1998 and 2003 were years with weak sea ice reduction, so they contrast strongly with 2006 and 2013. In the year 2011, sea ice concentration was minimal since 2005, but it was close to the average during the total period of observations (1979-2016).

**6. November precipitation anomalies (upper panel) and 1000-850 hPa specific humidity anomalies**

Data are shown for two years (1998, 2003) of weak sea ice reduction (left) and two years (2006, 2013) of strong sea ice reduction (right).  Anomaly is with respect to the November mean of ERA-Interim daily data during the period of 1979-2015. It is shown that anomalous precipitation and specific humidity are generally negative over the continent in years of weak sea ice reduction and are generally positive in years of strong sea ice reduction. This suggests that the likelihood of precipitation events increases with windows of marked autumn sea ice reduction in the Barents and Kara Seas.

**7. Patterns of anomalous precipitable water (kg/m^2^) in November 2003, 2006 and 2013.** Anomaly is with respect to the November mean during 2003-2014. This figure shows that the amount of precipitable water near the Yamal Peninsula was below normal in 2003 (a year of weak sea ice reduction), whereas it was above normal in 2013 (a year of strong sea ice reduction).


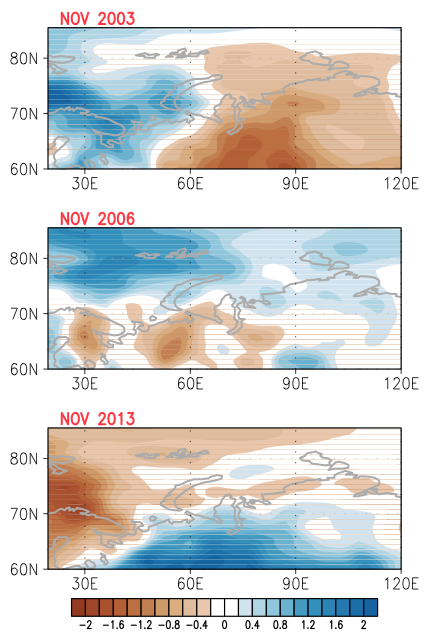


**8. Daily 1000-850 hPa specific humidity anomalies during November 1-10, 2006.**

Anomaly is with respect to the mean of November 2006. The daily pattern of anomalous specific humidity indicates that it has increased significantly around the Yamal Peninsula since 6 November, suggesting moisture convergence and ultimate precipitation events in this area.

**9.** **Daily 1000-850 hPa specific humidity anomalies during November 1-10, 2013.**

Anomaly is with respect to the mean of November 2013. The daily pattern of anomalous specific humidity indicates that it has increased significantly around the Yamal Peninsula since 7 November, suggesting moisture convergence and ultimate precipitation events in this area.

**10. Barents and Kara Sea meridional wind speeds and vectors from MERRA-2**

The figures for 2006 (top) and 2013 (bottom) are based on data from Modern-Era Retrospective analysis for Research and Applications, Version 2. Source: <https://gmao.gsfc.nasa.gov/reanalysis/MERRA-2/>

The graphics clearly show wind advection onto and across southern Yamal Peninsula from the BK Seas during the periods 6-7 November 2006 and 6-7 November 2013.

**
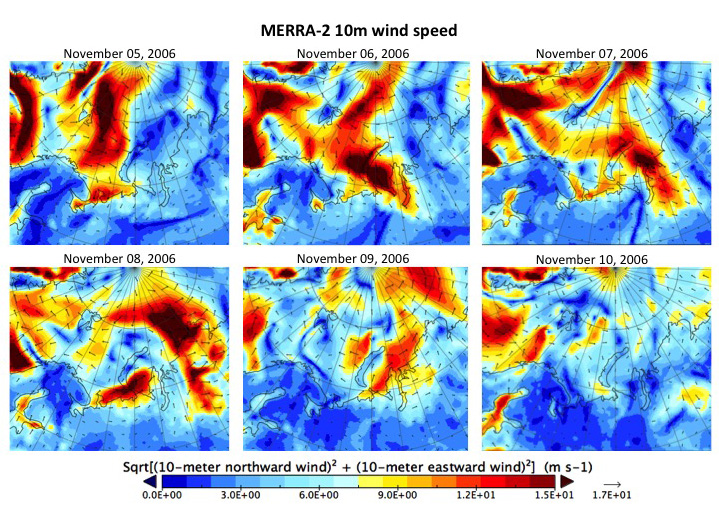
**

**
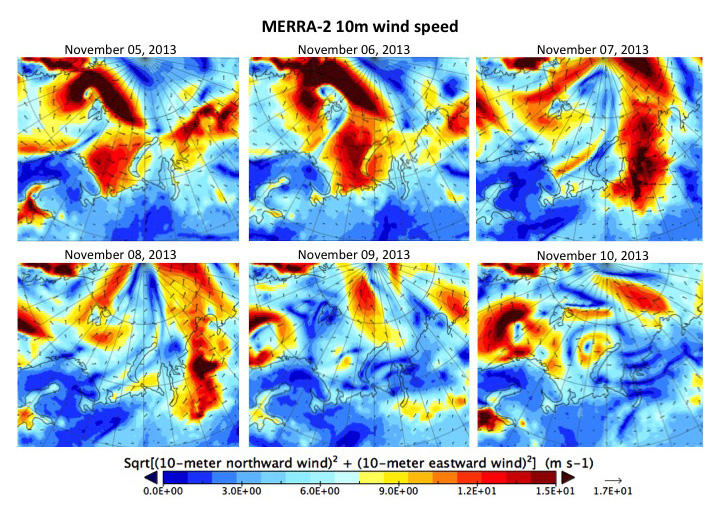
**
